# Single-cell m^6^A mapping in vivo using picoMeRIP–seq

**DOI:** 10.1038/s41587-023-01831-7

**Published:** 2023-06-22

**Authors:** Yanjiao Li, Yunhao Wang, Maria Vera-Rodriguez, Leif Christopher Lindeman, Linda Ellevog Skuggen, Erik M. K. Rasmussen, Ingunn Jermstad, Shaista Khan, Madeleine Fosslie, Trine Skuland, Marie Indahl, Sherif Khodeer, Eva Kristine Klemsdal, Kang-Xuan Jin, Knut Tomas Dalen, Peter Fedorcsak, Gareth D. Greggains, Mads Lerdrup, Arne Klungland, Kin Fai Au, John Arne Dahl

**Affiliations:** 1 Department of Microbiology, Oslo University Hospital, Rikshospitalet, Oslo, Norway.; 2 Department of Molecular Medicine, Institute of Basic Medical Sciences, University of Oslo, Oslo, Norway.; 3 Department of Biomedical Informatics, The Ohio State University, Columbus, OH, USA.; 4 Department of Computational Medicine and Bioinformatics, University of Michigan, Ann Arbor, MI, USA.; 5 Department of Reproductive Medicine, Oslo University Hospital, Rikshospitalet, Oslo, Norway.; 6 Faculty of Veterinary Medicine, Norwegian University of Life Sciences, Aas, Norway.; 7 Norwegian Transgenic Centre, Institute of Basic Medical Sciences, University of Oslo, Oslo, Norway.; 8 Division of Gynaecology and Obstetrics, Institute of Clinical Medicine, Faculty of Medicine, University of Oslo, Oslo, Norway.; 9 Center for Chromosome Stability, Department of Cellular and Molecular Medicine, Faculty of Health and Medical Sciences, University of Copenhagen, Copenhagen, Denmark.; 10 Department of Microbiology, Institute of Clinical Medicine, Faculty of Medicine, University of Oslo, Oslo, Norway.; 11 Biomedical Informatics Shared Resources, The Ohio State University, Columbus, OH, USA.; 12 Present address: KU Leuven-University of Leuven, Department of Development and Regeneration, Leuven Institute for Single-Cell Omics (LISCO), Leuven Stem Cell Institute, Leuven, Belgium.; 13 These authors contributed equally: Yanjiao Li, Yunhao Wang.

## Abstract

Current *N*^6^-methyladenosine (m^6^A) mapping methods need large amounts of RNA or are limited to cultured cells. Through optimized sample recovery and signal-to-noise ratio, we developed picogram-scale m^6^A RNA immunoprecipitation and sequencing (picoMeRIP–seq) for studying m^6^A in vivo in single cells and scarce cell types using standard laboratory equipment. We benchmark m^6^A mapping on titrations of poly(A) RNA and embryonic stem cells and in single zebrafish zygotes, mouse oocytes and embryos.

*N*^6^-Methyladenosine (m^6^A) is the most prevalent endogenous modification of mRNAs in eukaryotes^1,2^. The m^6^A modification is involved in regulating post-transcriptional RNA processes, including splicing^3^, export^4^, stability^5^, turnover^6^ and translation^7^, and has key roles in cell differentiation and reprogramming^8^, gametogenesis^9^, embryogenesis^10^, stress response^11^, tumorigenesis^12^ and cellular integrity maintenance by silencing endogenous retrovirus-derived RNAs^13^.

Since the first publications of m^6^A mapping methods in 2012 (m^6^A RNA immunoprecipitation and sequencing (MeRIP–seq^14^) and m^6^A-seq^15^), several techniques have been developed: antibody-based PA-m^6^A-seq^16^, miCLIP^17^, m^6^A-CLIP^18^ and m^6^A-LAIC-seq^19^ and antibody-free DART-seq^20^, MAZTER-seq^21^, m^6^A-REF-seq^22^ and m^6^A-SEAL^23^. Immunoprecipitation (IP)-based methods do not provide single-nucleotide resolution or m^6^A stoichiometry but can estimate position based on RRACH motif and can be used for differential enrichment analysis with tools such as DESeq2. Mapping of m^6^A typically requires large amounts of input material. The lowest starting amount reported to date is 10 ng of total RNA using the DART-seq technique^20^, and recently, single-cell DART-seq was also demonstrated^24^. However, DART-seq requires APOBEC1-YTH expression in cells to induce C-to-U deamination at sites adjacent to m^6^A residues, thus limiting its application to cultured cells^24^. Despite these advances, there is still a need for highly sensitive and single-cell m^6^A mapping methods applicable to in vivo cell types.

To this end, we developed a sensitive picogram-scale MeRIP–seq (picoMeRIP–seq) method that is also suitable for single-cell MeRIP–seq (Fig. 1a) and benchmarked m^6^A mapping on titrations of mouse liver poly(A)-selected RNAs, spike-in control RNAs and mouse embryonic stem (mES) cells and in single zebrafish zygotes, single mouse oocytes and preimplantation embryos. First, we performed optimization of experimental parameters using mouse liver poly(A)-selected RNA and *Pdzd8* mRNA as a positive control and *Rdh10* mRNA as a negative control based on published data^15^. We assessed the effects of several experimental conditions on the signal-to-noise (S/N) ratio (Methods). We have previously shown that optimizing the S/N ratio is critical for successful downscaling of chromatin IP^25^, and we reasoned that it would be equally important for RNA IP. This is based on the rationale that when scaling down the amount of starting material, while the surfaces available for nonspecific binding of RNA (the surface of plastic tubes and paramagnetic beads) are kept consistent, this results in a relative increase in the carryover of nonspecifically bound RNA and hence a reduction in S/N ratio. When scaling down the input amount, the S/N ratio is improved by the following: (1) increasing the detergent (SDS) and salt (NaCl) concentrations and roughly vortexing rather than gently rotating head over tail, suggesting that chemically and physically stringent washing is able to remove more of the nonspecifically bound material (Fig. 1a, step 4, Extended Data Fig. 1a and Methods); (2) using low-binding tubes to reduce carryover of nonspecifically bound material at the plastic surface of the tube and to reduce loss of RNA (Fig. 1a, steps 1–5, Extended Data Fig. 1b and Methods) and (3) thoroughly assessing commercially available antibodies to m^6^A and finding that anti-m^6^A from Millipore has a superior S/N ratio (Fig. 1a, step 3, Extended Data Fig. 1c and Methods).

Furthermore, we established reliable RNA fragmentation by sonication (Fig. 1a, step 2, Extended Data Fig. 1d and Methods). Finally, conventional preparation of RNA libraries requires conversion of the RNA starting material to cDNA and uses either RNA or DNA adaptor ligation to the target RNA or DNA molecules, which has relatively low efficiency. For epitranscriptome-wide mapping, we tailored Takara Bio’s Switching Mechanism At the 5′ end of RNA Template (SMART) library preparation protocol to a single-tube procedure (Fig. 1a, step 5, Extended Data Fig. 1e and Methods) with an efficient and simple workflow to generate stranded Illumina sequencing-ready libraries in a few hours. After Illumina sequencing, the resulting data were analyzed with standard preprocessing, read mapping and filtering (Fig. 1a, steps 6–8). Peak calling was performed with a commonly used model-based method, MACS^26^, providing m^6^A peaks for further downstream analyses (Fig. 1a, steps 9 and 10, and Methods).

We assessed the performance of picoMeRIP–seq on a titration of input RNA amounts (10 ng, 1 ng and 100 pg of mouse liver poly(A)-selected RNA) and compared it to published data. picoMeRIP–seq generated consistent profiles between replicates and for reduced starting amounts and reproduced published data (Fig. 1b). A high degree of transcriptome-wide correlation of sequencing reads was observed between replicates and between different starting amounts (Pearson correlation coefficients of 0.82–0.96; Fig. 1c). On average, the numbers of m^6^A peaks called from different amounts of starting material were 11,895 (10 ng), 12,079 (1 ng) and 6,661 (100 pg) (Supplementary Table 1). Peak overlap between different starting amounts and between replicates was high and on par with, or better than, the overlap between replicates of published data from 3 μg of total RNA (Fig. 1d,e and Extended Data Fig. 2a)^27^. picoMeRIP–seq (10 ng) identified about 87% of previously published peaks from liver RNA (3 μg of total RNA)^27^, supporting the reliability of our method (Fig. 1d). Furthermore, we validated the specificity of our method by showing that de novo motif analysis of data obtained from 10 ng, 1 ng and 100 pg of mouse liver RNA all identified the well-known m^6^A motif RRACH as the most significantly enriched motif (Extended Data Fig. 2b)^15,27^, and around 96% of m^6^A peaks from each sample had the RRACH motif (Supplementary Table 1). Last, mouse liver picoMeRIP–seq data presented clear m^6^A enrichment at the vicinity of the stop codon (Extended Data Fig. 2c), consistent with previous reports on m^6^A distribution^15,27^.

Next, we assessed the effect of a key computational analysis parameter (the *q* value reported by MACS) on the reliability of identified m^6^A peaks using the following four evaluation factors: (1) fraction of peaks with a RRACH motif, (2) fraction of peaks that are located in either the stop codon or 3’ untranslated region (UTR), (3) fraction of peaks identified in two biological replicates and (4) fraction of peaks identified by picoMeRIP–seq and previously published work using 3 μg of RNA^27^. As expected, the number of identified peaks decreased when increasing the stringency of the statistical significance cutoff (that is, lower *q* value) for peak calling, and the effect of this was comparable between picoMeRIP–seq data from 10 ng, 1 ng and 100 pg and published data (Extended Data Fig. 3a). Of note, the minor effect of *q* value cutoffs ranging from <0.05 to <1 × 10^−100^ on the four evaluation factors listed above supported robustness of the picoMeRIP–seq data (Extended Data Fig. 3b-e). Furthermore, both for published data and for picoMeRIP–seq data, we observed that peaks supported by two biological replicates had higher fractions of RRACH motifs and higher fractions of m^6^A peaks with 3′-end transcript occupancy than peaks only supported by one biological replicate (Extended Data Fig. 3f,g).

Thereafter, we performed further experimental assessments of the level of specificity and background of picoMeRIP. To assess the quantitative performance of picoMeRIP, two control RNAs with (Gaussia luciferase (GLuc)) and without (Cypridina luciferase (CLuc)) m^6^A modifications were mixed at different ratios to obtain five samples with different methylation levels (100%, 80%, 50%, 20% and 0%) that were used for picoMeRIP–quantitative PCR (picoMeRIP–qPCR; Extended Data Fig. 4a). We achieved high agreement (*R* = 0.99) between the expected m^6^A levels and the experimentally observed m^6^A levels (Extended Data Fig. 4b). Furthermore, we spiked in the two control RNAs into mouse liver mRNA samples at a 1:1 ratio (GLuc:CLuc) and performed picoMeRIP. qPCR analysis showed high m^6^A signal compared to unmodified background for both spike-in controls and for previously validated m^6^A-positive (*Pdzd8*) and m^6^A-negative (*Rdh10*) liver transcripts (Extended Data Fig. 4c). Next, we compared the number of m^6^A peaks and peak signal strength of picoMeRIP–seq from wild-type (WT) and METTL3-deficient mES cells, including spiked-in control RNAs. We made use of a published *Mettl3*-knockout (KO) mES cell line^8^ and confirmed the absence of the METTL3 m^6^A writer protein by western blotting (Extended Data Fig. 4d). To identify m^6^A peaks specific to either WT or KO mES cells, we performed picoMeRIP–seq on (1) mouse liver mRNA, (2) mouse liver mRNA and WT mES cell mRNA added in a 1:1 ratio and (3) mouse liver mRNA and KO mES cell mRNA added in a 1:1 ratio (Extended Data Fig. 4e). This allowed for quantitative comparison of m^6^A signal between WT and METTL3-deficient mES cells. We identified 7,404 peaks specific to WT and only 1,915 peaks specific to METTL3-deficient mES cells (Extended Data Fig. 4f) and found that METTL3-deficient mES cell-specific peaks showed significantly lower m^6^A signal than WT-specific peaks (Extended Data Fig. 4g). These results are in agreement with previous reports demonstrating that METTL3 deficiency leads to incomplete removal of m^6^A methylation activity^10^. In parallel, assessment of the m^6^A-modified GLuc and unmodified CLuc control RNAs spiked into the samples showed low false discovery rates (Extended Data Fig. 4h). Together, these data support high specificity and utility of picoMeRIP–seq in m^6^A detection.

Next, we applied picoMeRIP–seq to single zebrafish zygotes for proof-of-principle single-cell m^6^A profiling. With the aim of starting from intact single cells and reducing loss as much as possible, we combined cell lysis and removal of both rRNA and DNA into a one-tube procedure (Fig. 1a, step 1, and Methods). Each single-cell experiment yielded from 195,976 to 641,234 (with an average of 400,079) uniquely aligned and deduplicated read pairs (Supplementary Table 1). We used picoMeRIP–seq data from pools of multiple zygotes as a reference. Genome browser assessment of picoMeRIP–seq data from single zygotes showed m^6^A profiles similar to those obtained with pools of zygotes (Fig. 1f). All metagene profiles of m^6^A enrichment displayed a clear enrichment at the vicinity of the stop codon, in agreement with previous studies (Fig. 1g)^14,15,28^. We observed high transcriptome-wide correlation (Pearson correlation coefficient) between data from pools of zygotes (>0.98; Extended Data Fig. 5a,d), between data from single zygotes and pools of zygotes (0.83–0.88; Extended Data Fig. 5b,d) and between biological replicates of single zygotes (0.93–0.97; Extended Data Fig. 5c,d). The sensitivity of m^6^A peak detection improved with increasing numbers of reads (Extended Data Fig. 5e,f). By combining the data from five single-zygote experiments, we detected 8,516 peaks (Extended Data Fig. 5e). The numbers of detected peaks from single-zygote picoMeRIP–seq data were close to the expected maximum, as estimated by peak calling from randomly downsampled data from pools of 10 zygotes (Extended Data Fig. 5f). We observed a high fraction of peak overlap between picoMeRIP–seq data from single zygotes and data from pools of zygotes (89–95%) and a high fraction of m^6^A transcript overlap between picoMeRIP–seq data from single zygotes and published large-scale data^28^ (78–85%), indicating a high level of specificity (Extended Data Fig. 6a). Finally, as further support of specificity, all picoMeRIP–seq experiments, also from single zygotes, demonstrated RRACH as the most significantly enriched motif (Extended Data Fig. 6b).

To further demonstrate the versatility of our method, we applied picoMeRIP–seq to mES cells sorted by fluorescence-activated cell sorting. We showed a high level of reproducibility for selected loci (Extended Data Fig. 7a) and by transcriptome-wide correlation analysis of picoMeRIP–seq data from 1,000 to 10 cells (Extended Data Fig. 7b). Assessment of m^6^A peak overlap showed high reproducibility between three biological replicates for 1,000 cells and 100 cells and also good overlap between 1,000 cells and 10 cells (Extended Data Fig. 7c). Although increased variation between three replicates of ten cells was observed (Extended Data Fig. 7b) and peak overlap was reduced (Extended Data Fig. 7c), significantly enriched motifs in m^6^A peaks (Extended Data Fig. 7d) and metagene profiles of m^6^A enrichment (Extended Data Fig. 7e) from ten-cell experiments were in agreement with previous studies in mES cells^10^. In addition, we tried to apply picoMeRIP–seq to single mES cells but did not obtain sufficient libraries for sequencing.

Next, to benchmark picoMeRIP–seq, we applied it to single mouse germinal vesicle (GV)-stage oocytes, metaphase II (MII)-stage oocytes and single embryos at the zygote, two-cell, eight-cell and blastocyst stages to generate m^6^A maps. Each experiment resulted in 1.1 million–5.1 million uniquely mapped and deduplicated reads (Supplementary Table 1). High library complexity allowed for a resolution sufficient to assess m^6^A enrichment at individual loci in the transcriptomes of single oocytes and single embryos (Fig. 2a) and even allowed for peak calling, demonstrating that m^6^A marking is a distinct feature in single cells. On average, 12,901 m^6^A peaks were identified for each stage (Fig. 2b and Supplementary Table 1), and 4,677 (GV), 3,764 (MII), 3,555 (zygote), 4,389 (two-cell), 6,140 (eight-cell) and 6,104 (blastocyst) gene transcripts that were m^6^A modified were identified (Fig. 2b). Principal component analysis (PCA) revealed that single-oocyte and single-embryo m^6^A data contained sufficient information for accurate clustering according to cell identity and could even distinguish closely related oocyte and embryo stages (Fig. 2c), demonstrating the power of picoMeRIP–seq to identify cell populations. In the future, higher-throughput analysis will likely allow further evaluation of heterogeneity between single cells. Metagene profiles showed typical distribution of m^6^A enrichment near stop codons for all oocyte and embryo stages (Fig. 2d). The m^6^A consensus motif RRACH was identified from the called peaks for all picoMeRIP–seq experiments (Fig. 2e and Extended Data Fig. 8a), consistent with our bulk embryo work^29^. Enrichment at a certain genomic region compared to what would be expected by chance was assessed (Extended Data Fig. 8b). The stop codon and 3′UTR showed high m^6^A enrichment for all oocyte and embryo stages. However, GV oocytes presented with the highest enrichment, and the enrichment decreased in MII oocytes, zygotes and two-cell embryos before increasing again in eight-cell and blastocyst-stage embryos (Extended Data Fig. 8b). A similar trend was observed for the fraction of m^6^A peaks where, on average, 39% of GV oocyte m^6^A peaks mapped to the stop codon or 3′ UTR before dropping to 25%, 26% and 24% in MII oocytes, zygotes and two-cell embryos, respectively, and increasing again to 36–37% in eight-cell and blastocyst-stage embryos (Extended Data Fig. 8b). The m^6^A enrichment in protein-coding sequence (CDS) regions was relatively more stable across all oocyte and embryo stages, but the fraction of m^6^A peaks at CDS regions also reached the lowest levels in two-cell embryos. It is unclear whether the dynamics of m^6^A are associated with maternal transcript degradation and/or other maternal-to-zygotic transition events, which needs further exploration. Addressing m^6^A stoichiometry in oocytes and embryos would require the development of new technology. Gene ontology (GO) analysis suggested that m^6^A was marking transcripts of known biological relevance to early embryo development. All investigated oocyte and embryo stages showed that genes marked by m^6^A were enriched in GO terms involved in transcription-related processes (Extended Data Fig. 9a). Genes marked by m^6^A in certain oocyte and embryo stages were enriched in GO terms such as cell proliferation, apoptosis, RNA splicing and embryonic development. By contrast, genes not marked by m^6^A were enriched in GO terms involving basic metabolic processes.

As m^6^A is also present in noncoding RNAs, including retrotransposon-derived RNAs^13^, we assessed the capacity of single-oocyte/single-embryo picoMeRIP–seq to study retrotransposons. The enrichment of m^6^A across several types of retrotransposons in mouse GV and MII oocytes, zygotes and two-cell, eight-cell and blastocyst-stage embryos and other tissues was analyzed (Methods). The two retrotransposon subfamilies L1Md_A and L1Md_T of the LINE-1 family were frequently enriched for m^6^A signal in several different mouse tissues and mES cells but not in oocytes or before the eight-cell-stage (Extended Data Fig. 9b). Notably, the MTA subfamily, the evolutionarily younger member of the mammalian apparent LTR retrotransposons (MaLRs) family, was specifically enriched for m^6^A in GV oocytes to two-cell embryos (Extended Data Fig. 9c). Recent work supports dynamic m^6^A enrichment in the transcripts of transposable elements^29,30^. MaLR retrotransposable elements are mainly transcribed in oocytes and early embryos, with MTA sequences reported to have a notable expression in oocytes^31^. MTA sequences are maternally expressed, and their RNA is largely degraded around the major zygotic genome activation (ZGA; Extended Data Fig. 9c)^29,31^. One may speculate that m^6^A marking of MTA sequences could play a role in stage-specific expression through marking these sequences for maternal transcript degradation. Moreover, the high abundance of the ZGA-related retrotransposon MERVL^31^ was associated with frequent m^6^A marking in single two-cell embryos (Extended Data Fig. 9d), in agreement with bulk embryo work^29^, suggesting a potential regulatory role of m^6^A-marked MERVL transcripts in ZGA.

In summary, we have developed and benchmarked a picogram-scale method for small-scale and single-cell m^6^A mapping without the use of specialized equipment, allowing it to be readily adopted by many laboratories. We anticipate that picoMeRIP–seq will enable m^6^A profiling of scarce cell types from in vivo sources, such as biopsies from healthy and diseased tissues. With a sensitivity that allows for single-oocyte and single-embryo studies, picoMeRIP–seq will open up the study of the m^6^A landscape of human oocytes and preimplantation embryos in relation to fertility and developmental defects.

## Methods

### Ethics statement

Zebrafish and mouse experiments were approved by the Animal Research Committee of the Norwegian Food Safety Authority (Forsøksdyrforvaltningens tilsynsog søknadssystem (FOTS) IDs: 10898 and 24911). Experimental procedures conformed to the ARRIVE guidelines and were conducted in accordance with the ethical guidelines in Directive 2010/63/EU of the European Parliament on the protection of animals used for scientific purposes and Norwegian legislations.

### Antibodies and tubes

The following antibodies to m^6^A were used in the experiments: Millipore, ABE572; New England Biolabs (NEB), E1610S; Diagenode, C15200082-50; Synaptic Systems, 202003.

The following low-binding tubes were used in the experiments: Axygen Maxymum Recovery 1.5-ml low-bind tubes (VWR.no, 525-0230); Axygen Maxymum Recovery 0.6-ml low-bind tubes (VWR.no, 525-0229); Axygen Maxymum Recovery 0.2-ml low-bind tubes (VWR.no, 732-0679).

### Zebrafish zygotes, mouse liver, mES cells, mouse oocytes and embryo collection

Total RNA was extracted from 100 zebrafish zygotes using TRIzol reagent (Thermo Fisher Scientific) and eluted in 100 μl of RNase-free water. Then, 10-μl and 5-μl samples were taken for ten and five zygotes, respectively, and volumes were adjusted to 12 μl with nuclease-free water. Single zebrafish zygotes were manually picked and distributed into 12 μl of 1× lysis buffer (Takara). Finally, the samples were snap-frozen in liquid nitrogen and stored at −80 °C until further processing.

For Extended Data Fig. 7, mES cells (R1) were purchased from ATCC (SCRC-1011). Twelve microliters of 1× lysis buffer (Takara) was dispensed into each well of a 96-well PCR plate, and cells were sorted into each well according to the manufacturer’s instructions using a BD FACSMelody cell sorter (BD Biosciences). Plates were sealed with sealing films and immediately stored at −80 °C until further processing. *Mettl3*^−/−^ and WT control mES cell lines were gifted from S. Geula et al., Jacob H. Hanna laboratory, Weizmann Institute of Science^8^. *Mycoplasma* testing was performed on a regular basis, and all cell lines were free of *Mycoplasma.*

Mice were housed in individually ventilated cages (IVC, Scanbur) with a stable light/dark cycle (7:00 to 19:00), with 55 ± 5% relative humidity at 22 ± 2 °C with free access to water and standard rodent chow diet (2018S; 58 E% carbohydrate, 18 E% fat, 24 E%; Teklad Global 18% Protein Rodent Diet, Envigo). The presence of pathogens was monitored quarterly in accordance with the Federation of European Laboratory Animal Science Association guidelines. Animals were specific pathogen free according to the Federation of European Laboratory Animal Science Association recommendations (specific pathogen-free status).

To collect GV oocytes, 8-week-old C57BL6/N females were injected with 5 U of pregnant mare serum gonadotropin (PMSG), and 48 h after PMSG injection, ovaries were dissected, and oocytes were isolated by puncturing the follicles. The procedure was performed in M2 medium supplemented with 0.2 mM 3-isobutyl-1-methylxanthine (a cyclic nucleotide phosphodiesterase inhibitor; Sigma) to prevent the oocytes from further progress to GV breakdown. The cumulus cells were gently removed by pipetting, and the oocytes were briefly exposed to acidic Tyrode’s solution (Sigma) to remove the zona pellucida, followed by three washes in M2 medium.

To collect MII oocytes, 4- to 5-week-old C57BL6/N females were injected with 5 U of PMSG followed by 5 U of human chorionic gonadotropin (hCG) 45 h after PMSG injection. The oviducts were dissected 20–22 h later and transferred to a clean dish containing M2 medium (Sigma). The oviduct ampulla was identified under a stereomicroscope to isolate MII oocytes containing the cumulus mass. Oocytes were treated with 0.3 mg ml^−1^ hyaluronidase dissolved in M2 medium to remove the cumulus cells and were exposed to acidic Tyrode’s solution (Sigma) for a few seconds to remove the zona pellucida. Finally, MII oocytes were washed in M2 medium.

To collect early embryos, female mice were superovulated by hormone injection (5 U of PMSG followed by 5 U of hCG 45 h later) and transferred to cages with C57BL/6N males (8 weeks old) for mating. At 27–28 h (zygote), 39–43 h (two cell), 68–70 h (eight cell) and 92–94 h (blastocyst) after hCG administration, female mice were killed by cervical dislocation. Embryos were flushed from the reproductive tract into HEPES-buffered CZB medium and transferred to acidic Tyrode’s solution (Sigma) for a few seconds to remove the zona pellucida, followed by three washes in M2 medium.

The mouse oocytes and embryos were manually picked and sorted into 12 μl of 1× lysis buffer (Takara). The samples were snap-frozen in liquid nitrogen and stored at −80 °C until further use.

Total RNA of C57BL/6N mouse livers was extracted using TRIzol reagent. Poly(A)^+^ RNA was selected twice with a Dynabeads mRNA purification kit (Thermo Fisher Scientific). Identification of ribosomal RNA contamination was conducted using an Agilent 2100 Bioanalyzer according to the user manual.

### Real-time qPCR

cDNA was synthesized from m^6^A-immunoprecipitated RNA using SuperScript VILO master mix (Thermo Fisher Scientific), and real-time qPCR was conducted using Fast SYBR Green master mix (Thermo Fisher Scientific) following the manufacturer’s protocol.

The following *Pdzd8* and *Rdh10* primer sequences were used for real-time qPCR.

Positive m^6^A control *Pdzd8*:

Forward primer, 5′-GTGGTTCTCTCATAGGACATAAAG-3′

Reverse primer, 5′-CAAAGCCAGTTATCAATACAGTCA-3′

Negative m^6^A control *Rdh10*:

Forward primer, 5′-AGTGTAGTGCTCTGTTGTGT-3′

Reverse primer, 5′-CGCTGATCTCAAACTGACATC-3′

To calculate the S/N ratio, the following formula was used:

$$\text{S/N}\;\text{ratio}=\;[\;2^{\left(C_{t}\;\text{input}\;(Pdzd8\;(\text{corrected))}-C_{t}\;\text{IP}\;(Pdzd8)\right)}\;]\;∕\;[\;2^{\left(C_{t}\;\text{input}\;(Rdh10\;(\text{corrected}))-C_{t}\;\text{IP}\;(Rdh10)\right)}\;].$$

$C_{t}$ (cycle threshold) input (corrected) = ($C_{t}$ input – log_2_ (10)). We subtract log_2_ (10) when the input represents 1/10th of the amount used for RNA immunoprecipitation. This is in order to correct for the difference in starting amount used for the input, and is only applied if using a different amount of starting material for the input as compared to the RNA IP. When the same amount of starting material is used for both the input and the RNA IP, there will be no correction for the input amount.

### Single-cell picoMeRIP–seq

The following procedures were performed in a UV decontaminated LAF bench.

#### rRNA and DNA depletion.

For single-tube rRNA and DNA depletion, we performed rRNA depletion using an NEBNext rRNA depletion kit (NEB) with some modifications to the user manual. Specifically, 3 μl of the RNA/probe master mix was added to a 12-μl sample, which was then subjected to a temperature ramp from 95 °C for 2 min to 22 °C at a rate of −0.1 °C s^−1^, followed by a 5-min hold at 22 °C. Next, 5 μl of the RNase H reaction mix was added to the samples and incubated at 37 °C for 30 min, after which 30 μl of DNase I digestion mix was added and incubated at 37 °C for an additional 30 min. The resulting samples were purified using 2.2× volume of RNAClean XP beads, washed twice with 80% freshly prepared ethanol and eluted with 78 μl of nuclease-free water. Finally, 2 μl of RiboLock RNase inhibitor (40 U μl^−1^) was added to the sample to prevent RNA degradation, resulting in a sample volume of 80 μl.

#### RNA fragmentation by sonication.

A UP100H Ultrasonic Processor (Hielscher) with a 2-mm probe was used to sonicate the samples, using pulse settings with 0.5-s cycles and 27% power. The samples underwent *n* × 30 s sonication cycles, with 30 s of sonication followed by 30 s on ice for each cycle. The numbers (*n*) of sonication cycles used in this study for different amounts of input were optimized and can be found in Supplementary Table 1. For mouse liver samples, 10 ng and 100 pg were used to construct input libraries after sonication. RNA from pools of zygotes was used for input controls for single zebrafish zygotes (Supplementary Table 1). In the case of single mouse oocytes and early embryos, 10% of multiple oocyte/embryo RNA was removed and served as input control (Supplementary Table 1). To the samples consisting of 80 μl, 20 μl of 5× IP buffer (50 mM Tris-HCl (pH 7.5), 750 mM NaCl, 0.5% (vol/vol) NP-40 and 5 U μl^−1^ RiboLock RNase inhibitor) was added to make a final volume of 100 μl for sonication.

#### Antibody–bead incubation.

Before use, Dynabeads (Invitrogen) were washed by taking 20 μl of beads and washing them twice with 1× IP buffer (200 μl of 5× IP buffer supplemented with 800 μl of nuclease-free water) by vortexing, quickly centrifuging on a MiniGalaxy and placing on a magnetic rack before discarding the supernatant. In a separate tube, the antibody was diluted by taking 4 μl of anti-m^6^A, 16 μl of 5× IP buffer and 60 μl of nuclease-free water and mixing by gentle vortexing. The antibody-containing solution was added to the washed beads, and the antibody–beads were incubated overnight with head-over-tail rotation on a HulaMixer at 4 °C (40 r.p.m.). After conducting antibody testing and comparison experiments, anti-m^6^A from Millipore (ABE572) was selected for use in all subsequent experiments.

#### IP and washes.

Antibody-coated beads were captured on the tube wall in a magnetic rack. The supernatant from the antibody–bead incubation was discarded. Antibody-coated beads were washed twice with 200 μl of 1× IP buffer by vortexing (four times for 5 s each) to remove unbound antibodies that would otherwise compete for binding to the epitope. At the end of the second wash, the antibody-coated beads were transferred to 0.2-ml PCR tubes. From 200 μl, a volume of 10 μl of homogenous antibody-coated bead solution was transferred to each PCR tube. The tubes were quickly centrifuged on a MiniGalaxy and placed in a magnetic rack for at least 2 min or until the solution became clear. After removing the supernatant, 100 μl of sonicated sample RNA solution was added to each antibody–bead-containing tube, and the samples were incubated with head-over-tail rotation on a HulaMixer at 4 °C for 2 h (40 r.p.m.). Tubes were quickly centrifuged on a MiniGalaxy and placed in a magnetic rack. The supernatant was removed, and the RNA–antibody–bead complexes were washed four times in the following solutions, quickly spun and placed in a magnetic rack in between washes: washed once with ice-cold medium-stringency RIPA buffer (10 mM Tris-HCl (pH 8.0), 300 mM NaCl, 1 mM EDTA, 0.5 mM EGTA, 1% (vol/vol) Triton X-100, 0.2% (vol/vol) SDS and 0.1% (vol/vol) sodium deoxycholate), washed twice with ice-cold high-stringency RIPA buffer (10 mM Tris-HCl (pH 8.0), 350 mM NaCl, 1 mM EDTA, 0.5 mM EGTA, 1% (vol/vol) Triton X-100, 0.23% (vol/vol) SDS and 0.1% (vol/vol) sodium deoxycholate) and washed once with ice-cold medium-stringency RIPA buffer. After the four washes, tubes were quickly spun and placed in a magnetic rack, and the supernatant was discarded. The RNA–antibody–bead complexes were then resuspended in 100 μl of 1× IP buffer and incubated for 5 min. The samples were then quickly spun and placed in a magnetic rack, and the supernatant was removed. The RNA–antibody–bead complexes were resuspended in 147.9 μl of elution buffer (5 mM Tris-HCl (pH 7.5), 1 mM EDTA, 0.05 % (vol/vol) SDS and 1 U μl^−1^ RiboLock RNase inhibitor). Proteinase K (2.1 μl; NEB) was added to each tube, and tubes were then incubated on a Thermomixer at 1,200 r.p.m. and 55 °C for 1.5 h. After incubation, the tubes were briefly centrifuged and incubated further on a Thermomixer at 80 °C for 20 min to inactivate the Proteinase K. The samples were then placed in a magnetic rack for 2–3 min, and the supernatant containing the m^6^A-immunoprecipitated RNA was transferred to a new 1.5-ml low-binding tube. The remaining beads were resuspended again in 147.9 μl of elution buffer, and 2.1 μl of Proteinase K was added. The samples were placed immediately in a Thermomixer at 1,200 r.p.m. and 55 °C for 5 min, followed by inactivation of Proteinase K on a Thermomixer at 80 °C for 20 min. The tubes were then placed back in a magnetic rack for 2–3 min, and the supernatant was collected and pooled with the first supernatant in the same 1.5-ml low-binding tube to recover as much of the m^6^A-immunoprecipitated RNA as possible, resulting in a total volume of about 300 μl.

#### Ethanol precipitation.

For both input and immunoprecipitated RNA samples, nuclease-free water was added to each tube to result in a final volume of 400 μl. Next, 40 μl of 3 M sodium acetate (pH 5.2; Thermo Fisher Scientific) and 10 μl of linear acrylamide 5 mg μl^−1^ (Thermo Fisher Scientific) were added, followed by 1,000 μl of ice-cold 100% ethanol. The samples were vigorously vortexed without centrifugation or spinning and immediately placed at −80 °C for at least 2 h or overnight until completely frozen. Samples were recovered from −80 °C and allowed to briefly thaw on ice, and it was visually confirmed that all samples had thawed before starting centrifugation. The samples were centrifuged at 20,000*g* at 4 °C for 15 min, and the supernatant was carefully removed without disturbing the visible pellet. The pellet was then washed twice with 1 ml of ice-cold 75% ethanol. For washes, 75% ethanol was added, and the tube was gently vortexed until the pellet detached from the bottom; centrifugation was repeated as described above. After the last wash, as much as possible of the supernatant was removed, the tube lid was left open until all ethanol had evaporated, and the dried pellet was resuspended in 7 μl of nuclease-free water.

#### Library preparation and sequencing.

With modifications to the manufacturer’s protocol, as described earlier, the SMART-Seq stranded kit (Takara, 634442) was used to construct sequencing libraries. For the fragmented input or immunoprecipitated RNA, we performed the protocol without the fragmentation step. After the first PCR amplification and following AMPure bead purification, we resuspended the beads by adding 46.5 μl of nuclease-free water and skipped the ribosomal cDNA depletion protocol in Section D. We then incubated the samples at room temperature for 5 min to allow time for rehydration and recovered 46 μl of supernatant from each sample. We continued following the protocol until completion. The libraries were assessed for quantity using KAPA library quantification kits (Roche), and size distribution was assessed using TapeStation D1000 ScreenTape (Agilent Technologies). In combination, this information provided for good estimation of pooling at equimolar ratios. The pooled libraries were sequenced on a NovaSeq system (Illumina) with 50-base pair (bp) paired-end mode.

#### Spike-in controls.

Spike-in control RNAs and qPCR primers are from the EpiMark *N*^6^-methyladenosine enrichment kit. Before adding the spike-in control RNAs to an RNA sample for picoMeRIP, each control RNA was diluted to 0.001 fmol μl^−1^, and 1 μl of the diluted control RNA was added. For the picoMeRIP–qPCR titration experiment, each control RNA was diluted to 1 fmol in 100 μl. The two control RNAs were then mixed together at the indicated ratio used for picoMeRIP–qPCR (Extended Data Fig. 4a).

### Western blotting

Western blotting was performed as previously described^32^. Total proteins were extracted using RIPA lysis buffer (Thermo Scientific, 89900) containing protease inhibitor cocktail (Sigma-Aldrich, P8340) and phenylmethylsulfonyl fluoride (Sigma-Aldrich, P7626). Protein samples were denatured and resolved by Bolt Bis-Tris Plus gels (Invitrogen). Separated proteins were transferred onto nitrocellulose membranes and detected with primary antibodies to METTL3 (Abcam, ab195352) and GAPDH (Abcam, ab125247). The following secondary antibodies were used: donkey anti-mouse horseradish peroxidase (HRP; Abcam, ab6820) and donkey anti-rabbit (HRP; Abcam, ab6802). Blots were developed by enhanced chemiluminescence (Thermo Fisher Scientific, 32209 and 34095) and scanned with a Bio-Rad ChemiDoc XRS+ system.

### Sequencing data processing, m^6^A peak identification and motif analysis

The code used for quality check, alignment and filtering of sequencing reads, identification of m^6^A peaks and m^6^A consensus motifs and abundance estimation of gene transcripts is available at GitHub (https://github.com/Augroup/MeRipBox).

Quality of raw sequencing reads was assessed using FastQC (v0.11.8; https://www.bioinformatics.babraham.ac.uk/projects/fastqc/) with the default parameters. After trimming sequencing adapters and low-quality bases with Cutadapt (v1.8.1)^33^ with the parameter ‘-q 20,20 -m 20 −max-n 0.01 −trim-n’, the clean read pairs were mapped to the reference genomes (mm10 for mouse and danRer11 for zebrafish) and reference sequences (for the two spike-in RNA controls, obtained from the manual for the EpiMark *N*^6^-methyladenosine enrichment kit) using HISAT2 (v2.1.0)^34^ with the parameter ‘−5 8 −no-mixed −no-discordant’. Multiply mapped read pairs (that is, more than one genomic locus per read pair as reported by HISAT2) were discarded. We further filtered out PCR duplicates by using SAMtools (v1.9)^35^ and read mates that overlapped with the genomic coordinates of ribosomal RNAs by using BEDTools (v2.28.0)^36^. These uniquely aligned, deduplicated and non-rRNA reads were used for m^6^A peak calling.

We identified m^6^A peaks using a model-based method called MACS (v2.1.2)^26^ with the mode ‘callpeak’, the parameter ‘−keep-dup all -B −nomodel −call-summits’ and estimated transcriptome sizes of ‘−gsize 242010196’ for mouse and ‘−gsize 117608789’ for zebrafish. The statistical significance cutoff for the identified m^6^A peaks was a *q* value of <0.05. The processed reads and identified m^6^A peaks are summarized in Supplementary Table 1.

Focusing on the 400-bp region where the center is the genomic coordinate of m^6^A peak summits reported by MACS2, we searched for consensus motifs by using Homer (v4.11.1)^37^ findMotifsGenome. pl with the parameter ‘−rna −len 5,6,7,8’. The genomic strand of m^6^A peak summits was deduced based on their overlap with the annotated transcripts. The *P* values for all motifs were calculated and reported by Homer under the assumptions described at the Homer website (http://homer.ucsd.edu/homer/motif/). For mouse ES cell, oocyte and embryo samples, the four or five base positions starting with the GGAC motif were plotted in the corresponding figure panels.

Based on the gene annotation libraries (Gencode vM23 for mouse and Ensembl v100 for zebrafish), gene transcript expression abundance (transcript per million (TPM)) of input samples was estimated using StringTie (v1.3.5)^38^ with the parameter ‘−e −A’.

### Read density pileup visualization, Pearson correlation, m^6^A signal and PCA

We further removed the unpaired read mate from the reads that were used for m^6^A peak calling. We made the genome coverage bigWig format files (bin size = 10 bp, normalized by reads per kilobase per million reads (RPKM)) using deepTools (v3.2.0)^39^ bamCoverage with the parameter ‘-bs 10 −normalizeUsing RPKM’. Based on these bigWig files, we plotted the distribution of read density along exonic regions of the selected transcripts, which had higher expression abundance (by input samples) than other transcripts of a gene, and also performed transcriptome-wide (exonic region) Pearson correlation analysis using deepTools multiBigwigSummary (‘bins’ mode and window size = 1 kilobase (kb), exonic region) and plotCorrelation.

Based on the bigWig files of input and IP samples, for each 10-bp bin, we defined the m^6^A signal as the log_2_ ratio of (IP RPKM + 1) over (input RPKM + 1) and performed PCAs for single mouse oocytes and embryo samples using deepTools multiBigwigSummary (‘bins’ mode and window size = 1 kb, exonic region) and plotPCA.

### m^6^A peak annotation and m^6^A gene definition

Using BEDTools intersect, all m^6^A peaks were assigned a genomic feature by looking at the overlap between the peak summit and annotated genomic features (Gencode vM23 for mouse and Ensembl v100 for zebrafish), including (1) stop codon, which was defined as the region from the upstream 200 bp to downstream 200 bp surrounding the annotated stop codon, (2) 3′ UTR, (3) 5′ UTR, (4) CDS, (5) exon, (6) intron and (7) intergenic. If the peak summit of an m^6^A peak overlapped with more than one genomic feature, we chose only the one with the following order of priority: stop codon, 3′ UTR, 5′ UTR, CDS, exon, intron and intergenic.

Using MetaPlotR^40^, we generated the metagene profiles of m^6^A peak summits along the 5′ UTR, CDS and 3′ UTR of protein-coding genes. For each gene, only one transcript/isoform with highest expression abundance was used for plotting.

We defined a gene as m^6^A modified/marked if any of its genomic features (stop codon, 3′ UTR, 5′ UTR, CDS, exon and intron) overlapped with ≥1 m^6^A peak summit.

### GO analyses

For each developmental stage of mouse oocytes and early embryos, we only chose an m^6^A gene if any of its genomic features (stop codon, 3′ UTR, 5′ UTR, CDS and exon) overlapped with ≥1 m^6^A peak summit in both biological replicates. The top 1,000 statistically significant (based on *P* values of assigned m^6^A peaks) m^6^A genes per developmental stage were used for GO analyses with the online tool DAVID (v6.8)^41^ using all mouse genes as the background. Only GO terms with a *P* value of <0.05 in the library ‘GOTERM_BP_DIRECT’ were selected for visualization. As a comparison, we also performed GO analyses for the 1,000 randomly sampled genes, which were expressed (TPM > 1) and did not overlap with any m^6^A peaks for each developmental stage.

### m^6^A enrichment on retrotransposon-derived RNAs

Mouse retrotransposon annotation was obtained on 5 March 2021 using the UCSC Table Browser with the settings ‘clade=Mammal, genome=Mouse, assembly=GRCm38/mm10, group=Variation and Repeats, track=RepeatMasker, table=rmsk’. For each retrotransposon subfamily, we used only the genomic locus/copy if (1) its length was ≥90% of the full-length reference consensus sequence and (2) it had <50% overlap percentage with exons of annotated genes.

For a given retrotransposon locus, the mean RPKM value (from input samples) across all genomic bins (size = 10 bp) overlapping with this locus was denoted as its expression value, and the mean m^6^A signal value across all genomic bins (size = 10 bp) overlapping with this locus was denoted as its m^6^A signal value. At each developmental stage of mouse oocytes and embryos, for each retrotransposon locus, the mean expression value and the mean m^6^A signal value across two biological replicates were calculated. For each retrotransposon subfamily, the fraction of loci with >0 m^6^A signal value was calculated.

## Extended Data

**Extended Data Fig. 1. ∣:**
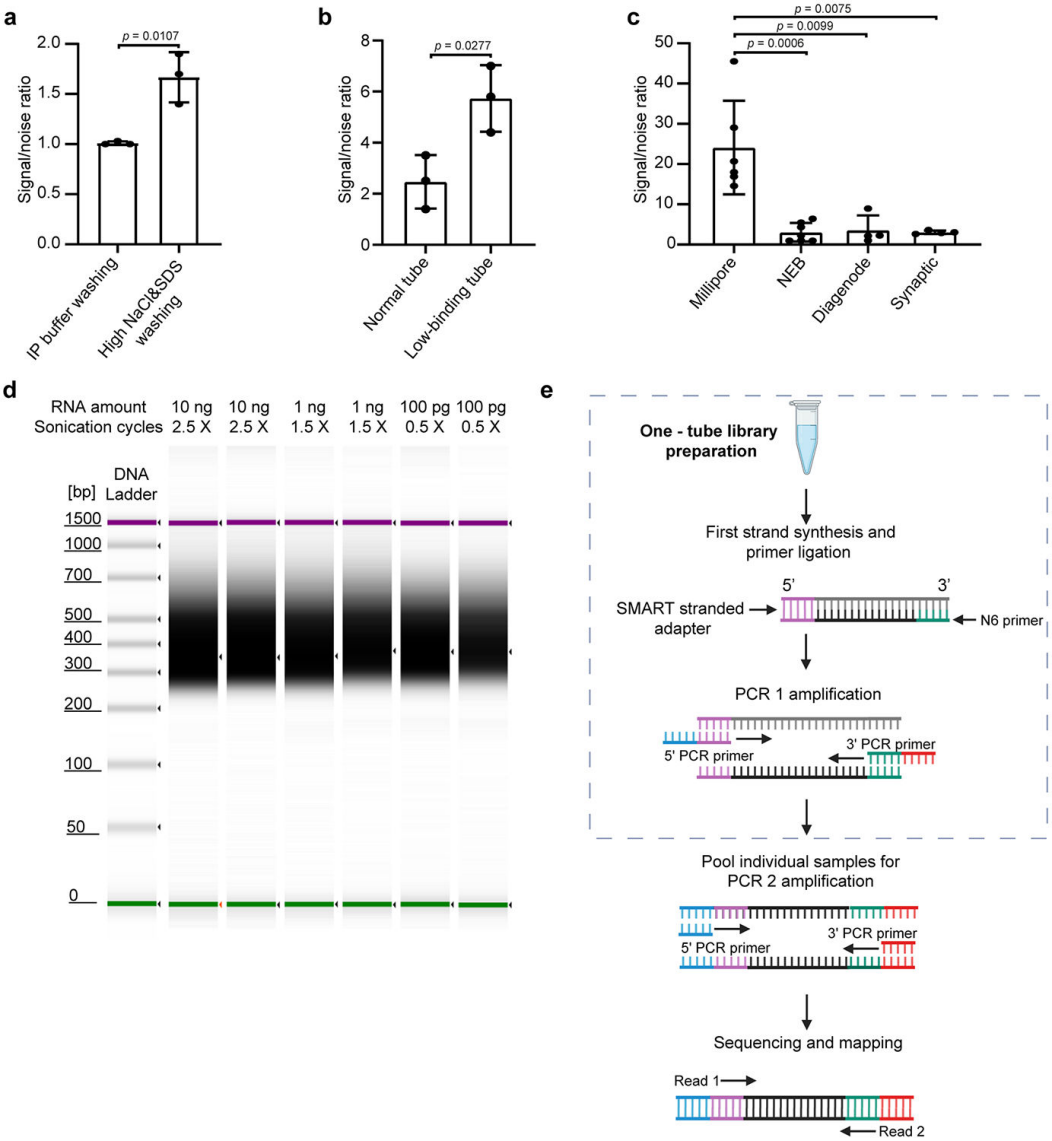
Optimization and development of picoMeRIP-seq. **a,** qPCR assessment of signal-to-noise ratio for the evaluation of the effect of increased stringency of the wash buffers. Mouse liver polyA selected RNA (10 ng) was used in these experiments. The previously validated m^6^A positive (*Pdzd8*) and negative (*Rdh10*) loci were used to calculate the signal-to-noise ratio (See Methods). Data are presented as mean ± standard deviation (SD), with n = 3 independent experiments for each experimental condition. Unpaired, two-tailed t-test was used. **b,** qPCR assessment of signal-to-noise ratio for the evaluation of the effect of low-binding tubes. Mouse liver polyA selected RNA (10 ng) was used in these experiments. Data are presented as mean ± SD values, with n = 3 independent experiments for each experimental condition. Unpaired, two-tailed t-test was used. **c,** qPCR assessment of signal-to-noise ratio for the optimized picoMeRIP method with different antibodies. Mouse liver polyA selected RNA (10 ng) was used in these experiments. Data are presented as mean ± SD values, with n = 6; 7; 4; 4 independent experiments for Millipore, NEB, Diagenode, Synaptic Systems antibodies, respectively. Unpaired, two-tailed t-test was used. **d,** Synthetic gel image from an Agilent 2100 Bioanalyzer electrophoreses run showing the size of sequencing libraries generated from picoMeRIP-seq of 10 ng, 1 ng and 100 pg mouse liver polyA selected RNA. Library preparation adds 139 bp to the size of the immunoprecipitated RNA fragments. The top line indicates the applied number of sonication cycles with the Hielscher UP100H sonicator. Each cycle is 30 seconds sonication plus 30 seconds on ice. **e,** Tailored SMART library preparation with a single-tube protocol to include as much as possible of the immunoprecipitated RNA. The SMART scN6 Primer (green) random primers allows the generation of cDNA from all immunoprecipitated RNA fragments. After the reverse transcription, the SMART Stranded Adapter (pink) will be linked with the cDNA product. Next, a first round of PCR amplification (PCR 1) adds full-length Illumina adapters, including barcodes. To minimize the number of samples to be processed downstream, individual samples can be pooled together for a second round of PCR amplification (PCR 2) using primers universal to all libraries. The final library is compatible with any Illumina sequencing platform. Created with BioRender.com.

**Extended Data Fig. 2 ∣:**
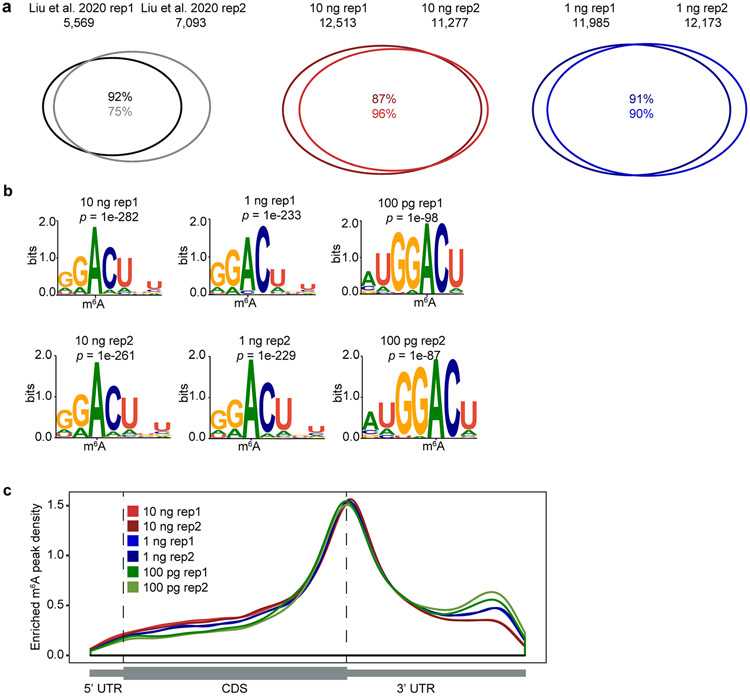
Assessment of picoMeRIP-seq using mouse liver RNA. **a,** Venn diagrams showing overlap of m^6^A peaks from two replicates of published work starting with 3 μg total RNA (Liu et al^27^), and for two replicates of picoMeRIP-seq experiments starting with either 10 ng or 1 ng of polyA RNA. **b,** Consensus motifs identified within m^6^A peaks from picoMeRIP-seq experiments with titration of the starting amount of polyA RNA. **c,** Metagene profiles showing the enrichment of m^6^A peaks along protein-coding gene transcripts.

**Extended Data Fig. 3 ∣:**
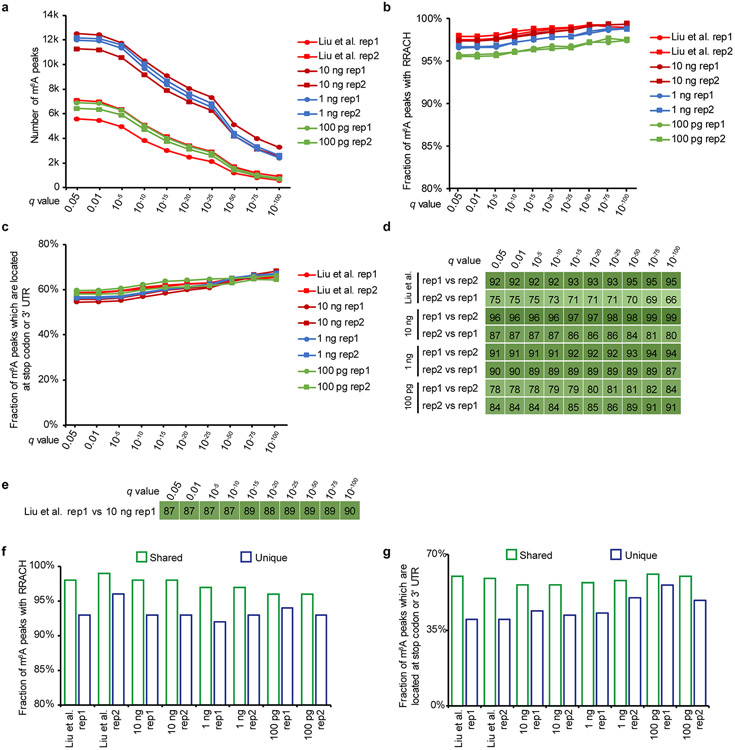
Evaluation of the reliability and robustness of picoMeRIP-seq by testing a range of different statistical significance cutoffs (that is, q value reported by MACS). **a,** Number of m^6^A peaks. **b,** Fraction of peaks with RRACH motif. **c,** Fraction of peaks which are located at stop codon or 3’ UTR. **d,** Fraction of peaks overlapping between two biological replicates. **e,** Fraction of peaks overlapping between picoMeRIP-seq (10 ng, biological replicate 1) and published data^27^ (3 μg RNA, biological replicate 1). **f,** Fraction of peaks with RRACH motif for the peaks that are identified in both biological replicates (shared) and the peaks that are only supported by one replicate (unique), respectively. **g,** Fraction of peaks that are located at stop codon or 3’ UTR for the shared peaks and unique peaks, respectively.

**Extended Data Fig. 4 ∣:**
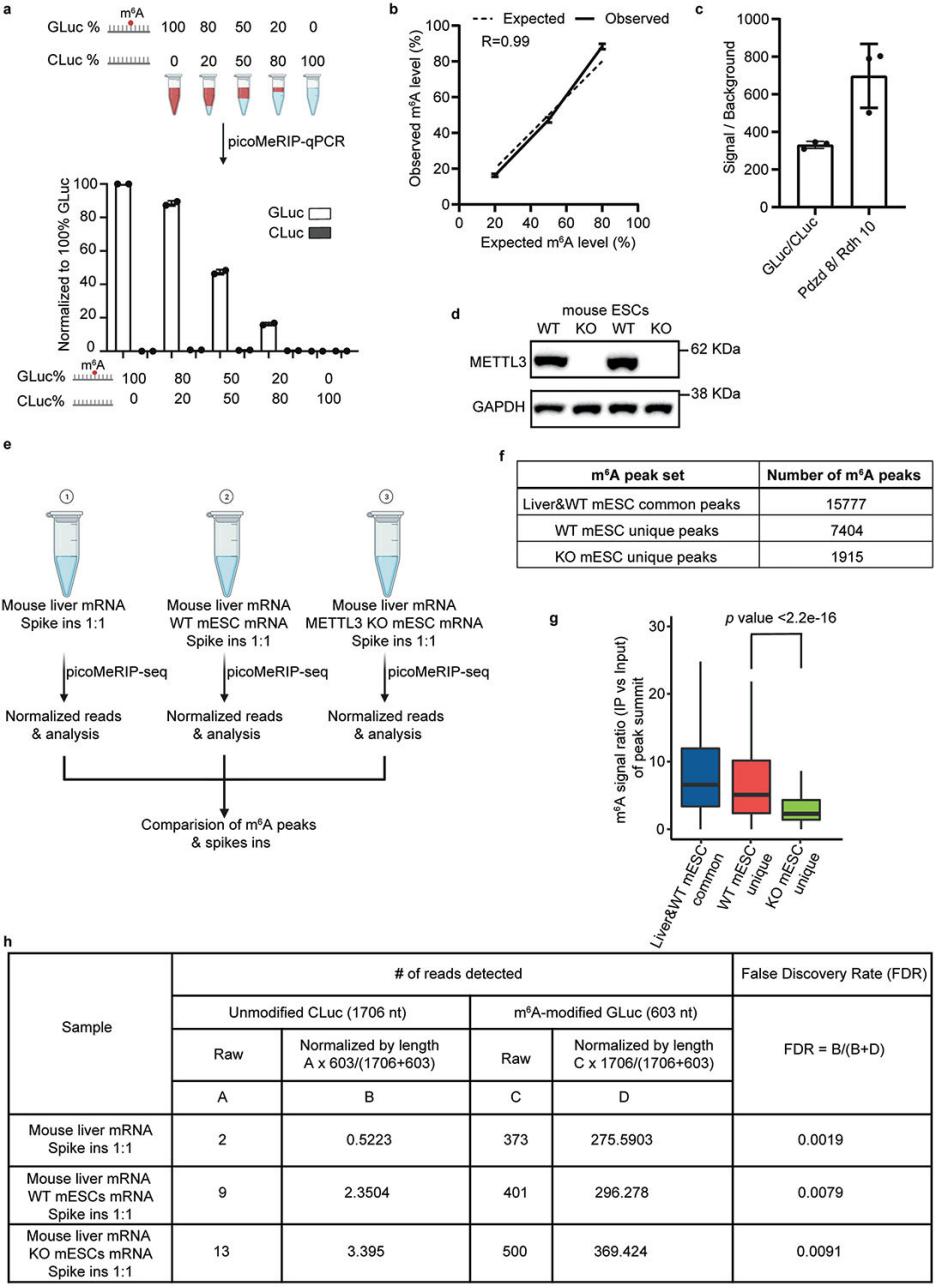
Characterization of specificity and background of picoMeRIP. **a,** Top panel, schematic illustration of experimental setup for titration of two control RNAs, m^6^A-modified GLuc, and unmodified CLuc, for picoMeRIP and qPCR assessment. Bottom panel, quantified immunoprecipitation levels of m^6^A-modified GLuc, and unmodified CLuc, after picoMeRIP. picoMeRIP signal was normalized to 100% GLuc. Data are presented as mean ± SD values, with n = 2 independent experiments for each experimental condition. Created with BioRender.com. **b,** Experimentally observed m^6^A levels of m^6^A-modified control RNA as compared to the expected ones based on the titration experiment described in panel a. Data are presented as mean ± SD values, with n = 2 independent experiments for each experimental condition. R is the correlation coefficient in regression analysis. **c,** qPCR assessment of picoMeRIP m^6^A signal over background with two control RNAs used as spike-ins, m^6^A-modified GLuc, and unmodified CLuc, and for the previously validated m^6^A positive (*Pdzd8*) and negative (*Rdh10*) liver transcripts. Data are presented as mean ± SD values, with n = 3 independent experiments for each experimental condition. **d,** Western blot of METTL3 and GAPDH for WT and *Mettl3* deficient (“knock-out” (KO)) mES cell lines. GAPDH was used as loading control. Two independent western blot experiments were performed with similar results. **e,** Schematic illustration of experimental setup to compare number of m^6^A peaks and peak signal strength of picoMeRIP-seq from WT and *Mettl3* deficient (KO) mES cells, including spiked-in control RNAs. Mouse liver mRNA and mES cell mRNA were added in a 1:1 ratio. Two control RNAs were used as spike-ins, m^6^A-modified GLuc, and unmodified CLuc, and added at a 1:1 ratio. Created with BioRender.com. **f,** Number of picoMeRIP-seq m^6^A peaks uniquely identified in WT and *Mettl3* deficient (KO) mES cells, and co-identified in mouse liver and WT mES cells. **g,** Box plots of m^6^A signal for comparison of picoMeRIP-seq peaks uniquely identified in WT and *Mettl3* deficient (KO) mES cells. Middle vertical line (bold) depicts the median, and the 25^th^ percentile to 75^th^ percentile is shown. Whiskers extend to the most extreme data point within 1.5 interquartile range of the quartiles. The *p* value was calculated using a Wilcoxon rank sum test (two-sided). The corresponding m^6^A peak numbers for each of three groups are shown in panel f. **h,** Assessment of false discovery rate of picoMeRIP-seq by spike-in m^6^A-modified GLuc, and unmodified Cluc control RNAs.

**Extended Data Fig. 5 ∣:**
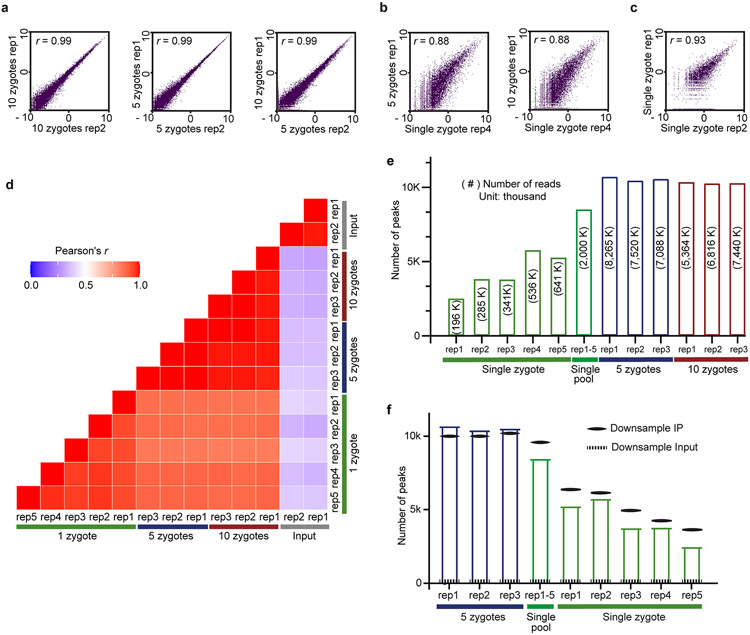
picoMeRIP-seq from single zebrafish zygotes. **a, b, c,** Scatter plots showing the correlation of picoMeRIP-seq experiments for (**a**) pooled zygotes; (**b**) pooled zygotes and single zygote; and (**c**) single zygote. The scatter plots are plotted using the natural log of read coverage (RPKM + 1). *r,* Pearson correlation coefficient. **d,** Heatmap comparing picoMeRIP-seq experiments from pooled zygotes and single zygote. *r*, Pearson correlation coefficient. **e,** Number of m^6^A peaks. Peak detection when combining the data from five single-zygote experiments is also shown. The numbers of uniquely mapped, deduplicated, non-rRNA-derived sequencing reads are shown inside the bars within parentheses. **f,** Number of m^6^A peaks called from different picoMeRIP-seq experiments from pools of zygotes and for single zygotes shown as full columns, as reference. Number of m^6^A peaks called from randomly down-sampled data from a picoMeRIP-seq experiments from a pool of ten zygotes is indicated with solid black lines. Down-sampling was performed to produce equal reads numbers as that originally yielded from each of the indicated experiments. The numbers of detected peaks from single-zygote picoMeRIP-seq is close to the expected maximum as estimated by peak calling from randomly down-sampled data from pools of ten zygotes. As a control, peak calling was also performed from randomly down-sampled data from input.

**Extended Data Fig. 6 ∣:**
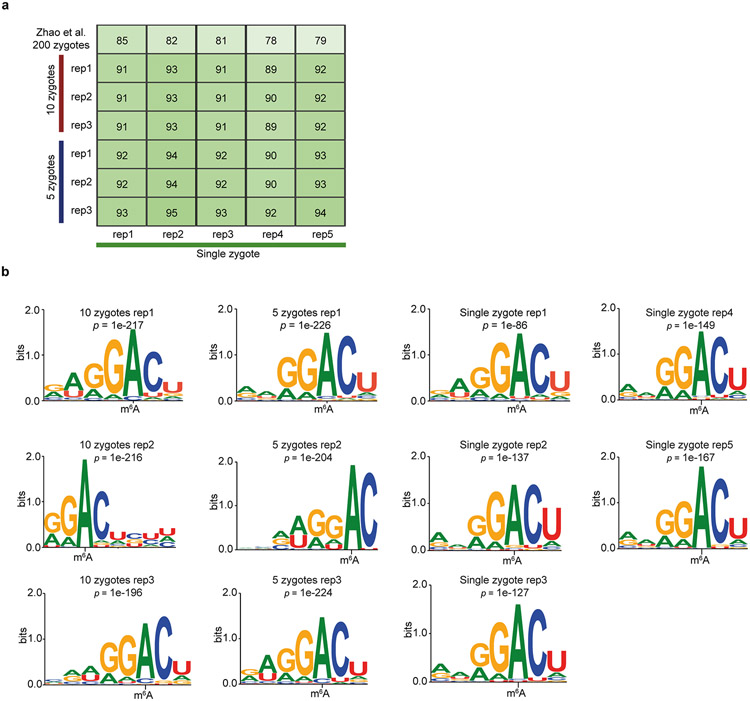
Comparison of picoMeRIP-seq from single zebrafish zygotes and from pools of zygotes. **a,** Fraction of overlap of m^6^A peaks between single-zygote picoMeRIP-seq experiments and picoMeRIP-seq from pools of zygotes. The topmost row is the fraction of overlap of m^6^A transcripts with previously published work (Zhao et al^28^, GEO accession: GSM2088167 and GSM2088177). **b,** Consensus motifs identified within m^6^A peaks from single-zygote picoMeRIP-seq experiments and for picoMeRIP-seq from pools of zygotes.

**Extended Data Fig. 7 ∣:**
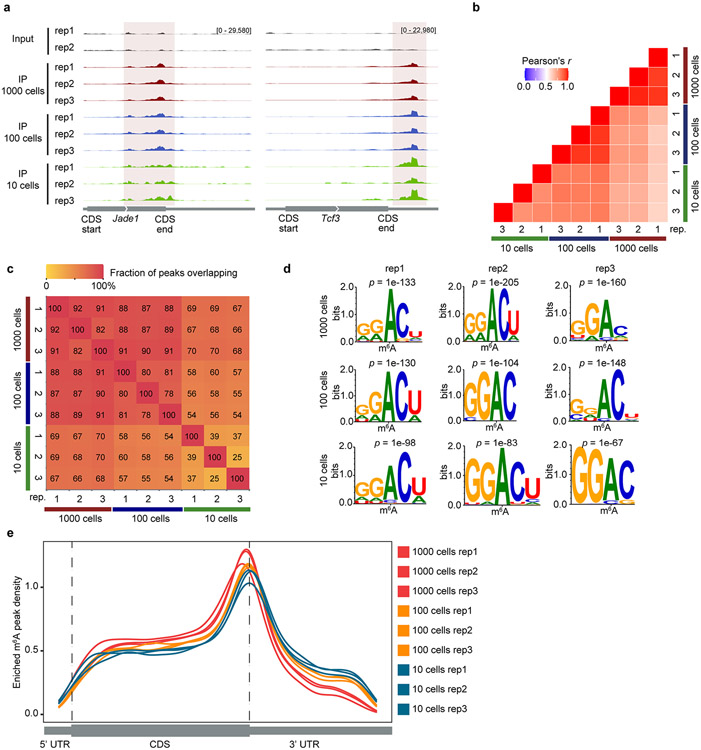
picoMeRIP-seq from FACS sorted mouse ES cells. **a**, Genome browser snap shots of two transcripts with m^6^A enrichment (transcript ID: ENSMUST00000026865.14 for *Jade1,* ENSMUST00000105344.7 for *Tcf3*). **b**, Transcriptome-wide correlation analyses (sequencing read coverage) between picoMeRIP-seq experiments from 1,000, 100 and 10 mouse ES cells. **c,** Heatmap showing fraction of overlap of m^6^A peaks between picoMeRIP-seq experiments with 1,000, 100 and 10 mouse ES cells. Peaks identified in the samples indicated at the bottom of the plot were used as the reference when calculating fraction of overlap of peaks from samples indicated at the left side of the plot. **d,** Consensus motifs identified within m^6^A peaks from picoMeRIP-seq experiments with 1,000, 100 and 10 mouse ES cells. **e,** Metagene profiles showing the enrichment of m^6^A peaks along protein-coding gene transcripts for 1,000, 100 and 10 mouse ES cells.

**Extended Data Fig. 8 ∣:**
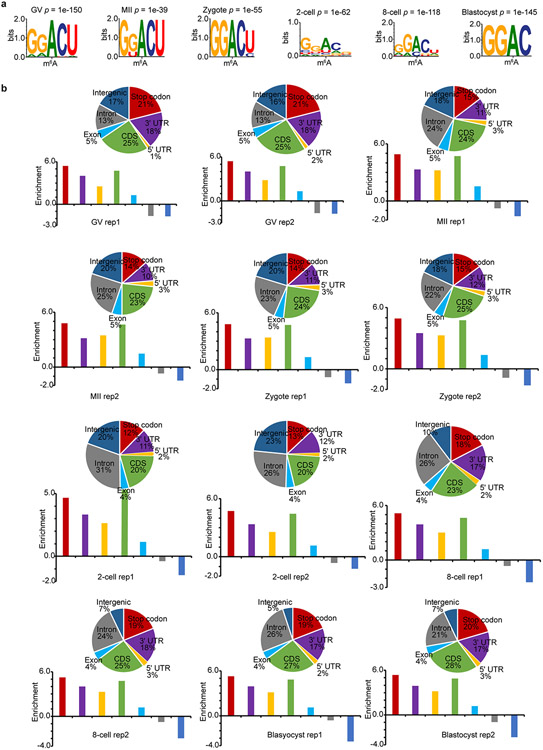
Consensus motifs and peak annotation from single mouse oocytes and preimplantation embryos. **a,** Consensus motifs identified within m^6^A peaks of biological replicate 2. **b,** Top, pie charts show genomic annotation of m^6^A peaks. Bottom, bar plots show the peak enrichment score, which was calculated as the log_2_ ratio of the observed over expected peak numbers.

**Extended Data Fig. 9 ∣:**
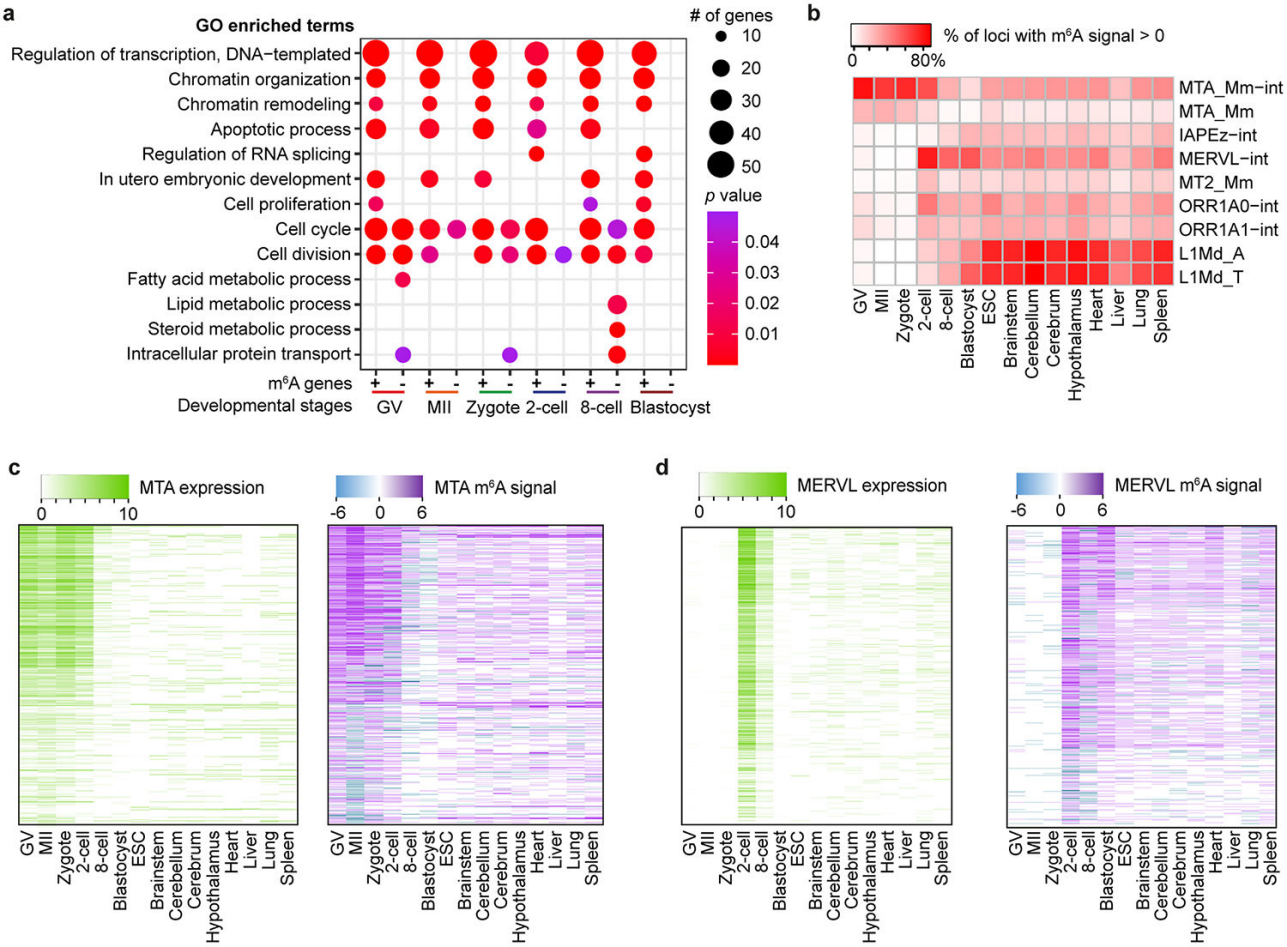
GO analysis and enrichment of m^6^A on retrotransposon-derived RNAs in mouse oocytes and embryos. **a,** Gene Ontology (GO) analysis for m^6^A-marked transcripts (+) and for transcripts not marked by m^6^A (−). The modified Fisher exact *p* values were (that is EASE score) reported by DAVID (see https://david.ncifcrf.gov/helps/functional_annotation. html). **b,** Enrichment of m^6^A in transcripts from selected retrotransposon subfamilies shown for mouse oocytes, embryos, ES cells (ESCs) and eight mouse tissues. MeRIP-seq data for mouse tissues are from Liu *et al.*^27^. **c, d,** Expression and m^6^A signal profiles across the internal sequences of MTA (**c**); and MERVL (**d**). The expression is RPKM calculated by deepTools (see Methods).

## Supplementary Material

Source Data Extended Data Fig. 4

Supplementary Table 1

## Figures and Tables

**Fig. 1 ∣:**
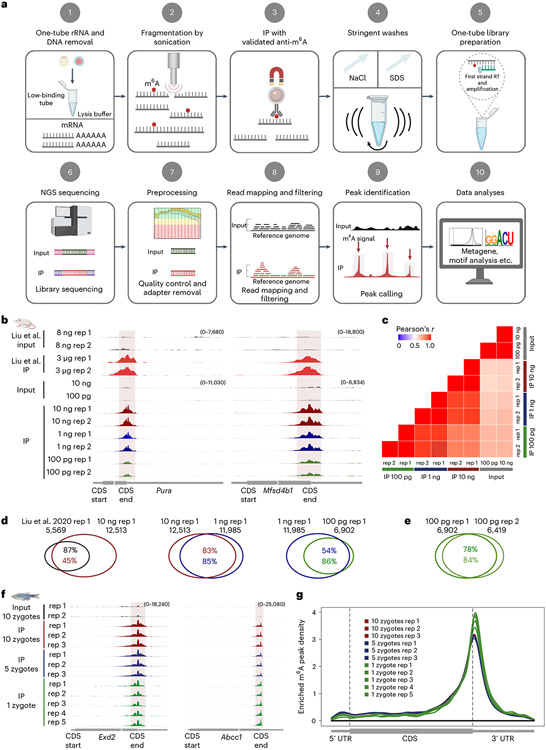
Development of picoMeRIP–seq. **a**, Schematic of the single-cell MeRIP–seq method and analysis pipeline; RT, reverse transcription; NGS, next-generation sequencing. Figure created with BioRender.com. **b**, Genome browser snapshots of two transcripts (transcript IDs: ENSMUST00000051301.5 for *Pura* and ENSMUST00000163705.2 for *Mfsd4b1*) harboring m^6^A enrichment near the stop codon. Tracks are shown for picoMeRIP–seq experiments with titration of the starting amount of poly(A)-selected RNA compared to published work starting with 3 μg of total RNA (Liu et al.^27^). **c**, Transcriptome-wide correlation analyses (sequencing read coverage). **d**, Venn diagrams showing overlap of m^6^A peaks from picoMeRIP–seq experiments and comparison to published work starting with 3 μg of total RNA (Liu et al^27^). **e**, Overlap of m^6^A peaks between two picoMeRIP–seq experiments from 100 pg of poly(A)-selected RNA. **f**, Genome browser snapshots of zebrafish zygote picoMeRIP–seq for two transcripts (transcript IDs: ENSDART00000111389 for *Exd2* and ENSDART00000161897 for *Abcc1*) harboring m^6^A enrichment near the stop codon. **g**, Metagene profiles for zebrafish zygote picoMeRIP–seq showing the enrichment of m^6^A peaks along protein-coding gene transcripts.

**Fig. 2 ∣:**
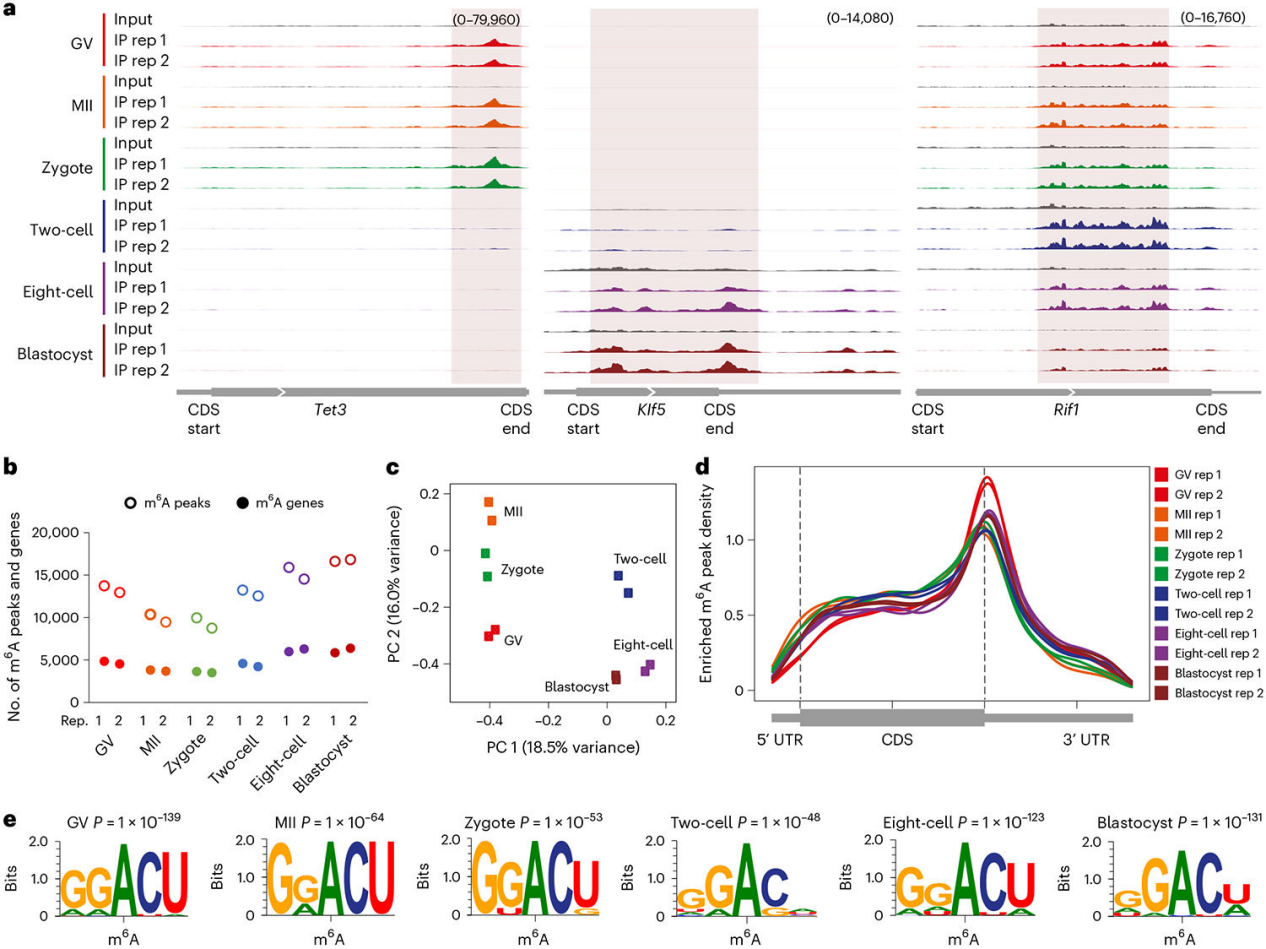
Profiling m^6^A methylation in single mouse oocytes and preimplantation embryos. **a**, Genome browser snapshots showing three examples of transcripts with dynamic m^6^A enrichment during mouse oocyte and embryo development (transcript IDs: ENSMUST00000186548.6 for *Tet3,* ENSMUST00000005279.7 for *Klf5* and ENSMUST00000112693.9 for *Rif1*). **b**, Number of m^6^A peaks and m^6^A-marked gene transcripts. **c**, PCA of m^6^A signal. **d**, Metagene profiles showing the enrichment of m^6^A peaks along protein-coding gene transcripts. **e**, Consensus motifs identified within m^6^A peaks identified in biological replicate 1.

## Data Availability

All sequencing data generated in this study are available in the Gene Expression Omnibus (GEO) under accession number GSE184893 (ref. 42). The m^6^A-seq data of eight mouse tissues were obtained from Genome Sequence Archive in BIG Data Center, Beijing Institute of Genomics (BIG), Chinese Academy of Sciences (http://bigd.big.ac.cn/gsa) under accession number CRA001962 (ref. 27). The processed m^6^A-seq dataset of bulk zebrafish zygotes was from GEO under accession numbers GSM2088167 and GSM2088177 (ref. 28). Source data are provided with this paper.

## Code availability

The major analysis scripts in this study were integrated into the bioinformatics pipeline MeRipBox, which is available at GitHub (https://github.com/Augroup/MeRipBox)^43^.
