# Supplementary figures and images for: Single-cell m6A mapping in vivo using picoMeRIP–seq
Source: Nat Biotechnol. Author manuscript; Available in PMC 2024 Apr 17. (PMC10739642; doi:10.1038/s41587-023-01831-7)

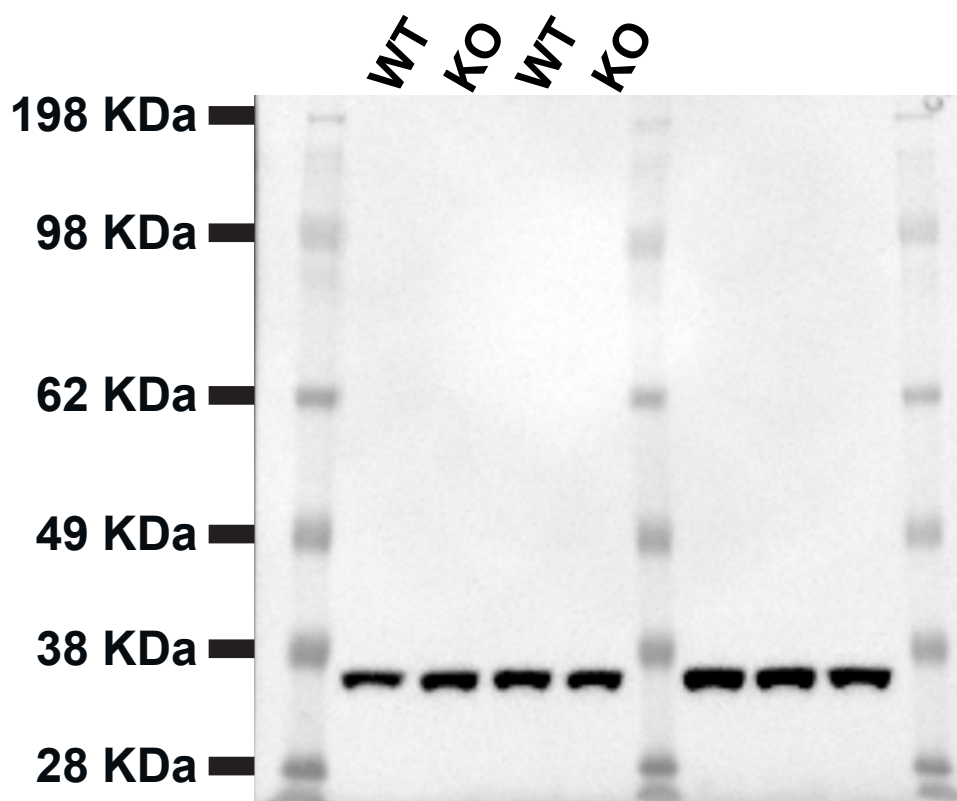

Raw blot: GAPDH

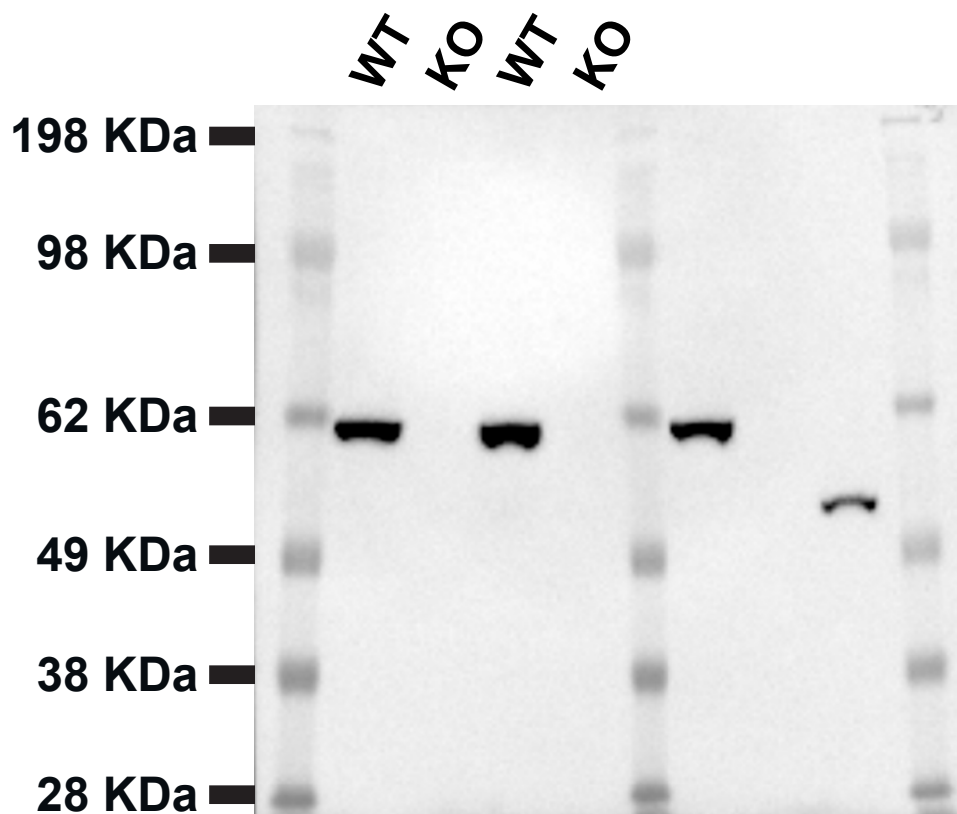

Raw blot: METTL3

Supplement: Source Data Extended Data Fig. 4 [file NIHMS1911563-supplement-Source_Data_Extended_Data_Fig__4.pdf]
