# Supplementary material for: Single-cell m6A mapping in vivo using picoMeRIP–seq
Source: Nat Biotechnol. 2023 Jun 22;42(4):591–6. doi: 10.1038/s41587-023-01831-7 (PMC10739642; doi:10.1038/s41587-023-01831-7)
Supplement: Supplementary file 1 [file 41587_2023_1831_MOESM1_ESM.pdf]

nature portfolio

Corresponding author(s): Arne Klungland, Kin Fai Au, John Arne Dahl

Last updated by author(s): May 5, 2023

Reporting Summary

Nature Portfolio wishes to improve the reproducibility of the work that we publish. This form provides structure for consistency and transparency in reporting. For further information on Nature Portfolio policies, see our Editorial Policies and the Editorial Policy Checklist.

Statistics

For all statistical analyses, confirm that the following items are present in the figure legend, table legend, main text, or Methods section.

n/a	Confirmed	
☐	☒	The exact sample size (*n*) for each experimental group/condition, given as a discrete number and unit of measurement
☐	☒	A statement on whether measurements were taken from distinct samples or whether the same sample was measured repeatedly
☐	☒	The statistical test(s) used AND whether they are one- or two-sided<br>*Only common tests should be described solely by name; describe more complex techniques in the Methods section.*
☒	☐	A description of all covariates tested
☒	☐	A description of any assumptions or corrections, such as tests of normality and adjustment for multiple comparisons
☐	☒	A full description of the statistical parameters including central tendency (e.g. means) or other basic estimates (e.g. regression coefficient) AND variation (e.g. standard deviation) or associated estimates of uncertainty (e.g. confidence intervals)
☐	☒	For null hypothesis testing, the test statistic (e.g. *F*, *t*, *r*) with confidence intervals, effect sizes, degrees of freedom and *P* value noted<br>*Give P values as exact values whenever suitable.*
☒	☐	For Bayesian analysis, information on the choice of priors and Markov chain Monte Carlo settings
☒	☐	For hierarchical and complex designs, identification of the appropriate level for tests and full reporting of outcomes
☐	☒	Estimates of effect sizes (e.g. Cohen's *d*, Pearson's *r*), indicating how they were calculated

Our web collection on statistics for biologists contains articles on many of the points above.

Software and code

Policy information about availability of computer code

Data collection	Sequencing was performed using Illumina platforms as described in Online Methods.<br>Mouse (version: mm10) and zebrafish (version: danRer11) reference genomes were obtained from UCSC Genome Browser.<br>Mouse gene annotation library (version: vM23) was obtained from GENCODE.<br>Zebrafish gene annotation (version: v100) was obtained from Ensembl.<br>Mouse retrotransposon annotation library was downloaded using UCSC Table Browser with the setting “clade=Mammal, genome=Mouse, assembly=GRCm38/mm10, group=Variation and Repeats, track=RepeatMasker, table=rmsk” on March 5, 2021.<br>Latest Biorender is available online for any computer. No download required.
Data analysis	FastQC (v0.11.8), Cutadapt (v1.8.1), HISAT2 (v2.1.0), SAMtools (v1.9), BEDTools (v2.28.0), StringTie (v1.3.5), deepTools (v3.2.0), MACS2 (v2.1.2), MetaPlotR (no version is indicated by the developers), Homer (v4.11.1), DAVID (v6.8), MeRipBox (v0.1), Biorender (an online tool, without specific version).

For manuscripts utilizing custom algorithms or software that are central to the research but not yet described in published literature, software must be made available to editors and reviewers. We strongly encourage code deposition in a community repository (e.g. GitHub). See the Nature Portfolio guidelines for submitting code & software for further information.

Data

Policy information about availability of data

All manuscripts must include a data availability statement. This statement should provide the following information, where applicable:

- Accession codes, unique identifiers, or web links for publicly available datasets
- A description of any restrictions on data availability
- For clinical datasets or third party data, please ensure that the statement adheres to our policy

All sequencing data generated in this study are available in the Gene Expression Omnibus (GEO) with accession number GSE184893. The m6A-seq data of eight mouse tissues were obtained from Genome Sequence Archive in BIG Data Center, Beijing Institute of Genomics (BIG), Chinese Academy of Sciences (http://bigd.big.ac.cn/gsa) under accession number CRA001962. The processed m6A-seq dataset of bulk zebrafish zygotes were from GEO under accession numbers GSM2088167 and GSM2088177.

Field-specific reporting

Please select the one below that is the best fit for your research. If you are not sure, read the appropriate sections before making your selection.

☒ Life sciences ☐ Behavioural & social sciences ☐ Ecological, evolutionary & environmental sciences

For a reference copy of the document with all sections, see nature.com/documents/nr-reporting-summary-flat.pdf

Life sciences study design

All studies must disclose on these points even when the disclosure is negative.

Sample size	No statistical methods were used to predetermine the sample size. This is a proof-of-concept study. Rather than focusing on high throughput, we provide a proof-of-concept study demonstrating that single-cell and single-embryo m6A profiling is achievable.
Data exclusions	No data was excluded in our analysis.
Replication	Each biological state is represented by multiple replicates, which whenever possible are visualized independently to give the reader full insight into the variation in the data. Number of replicates are indicated in figure legends.
Randomization	Samples from different biological stages were sequenced together in the same runs, and no run consisted of a large overweight of a single stage. We believe there was no need for further randomization for this study. All experiments to be compared were carried out under the same experimental conditions, except for when testing different parameters for methods optimization. For methods optimization, one parameter at the time was modified and assessed for each set of experiments, as indicated in all relevant figures and legends.
Blinding	Blind testing can be used when items are to be compared without influences from testers' expectations. We believe that this was not relevant for our MeRIP-seq study as data were analyzed with computational metrics and no human scoring involved. The authors were not blinded to group allocation. Blinding was not necessary for experimental design and implementation.

Reporting for specific materials, systems and methods

We require information from authors about some types of materials, experimental systems and methods used in many studies. Here, indicate whether each material, system or method listed is relevant to your study. If you are not sure if a list item applies to your research, read the appropriate section before selecting a response.

Materials & experimental systems

n/a	Involved in the study	
☐	☒	Antibodies
☐	☒	Eukaryotic cell lines
☒	☐	Palaeontology and archaeology
☐	☒	Animals and other organisms
☒	☐	Human research participants
☒	☐	Clinical data
☒	☐	Dual use research of concern

Methods

n/a	Involved in the study	
☒	☐	ChIP-seq
☒	☐	Flow cytometry
☒	☐	MRI-based neuroimaging

Antibodies

Antibodies used	The following antibodies to m6A were used in the experiments: Millipore, polyclonal, Cat # ABE572 (Lot # 3194595); NEB, monoclonal, Cat # E1610S (Lot # 10015190); Diagenode, monoclonal, Cat # C15200082-50 (Lot # 001); Synaptic Systems, polyclonal,

	Cat # 202003(lot# 201905/10). As described in the methods section, a pre-mixed antibody mix (4 µl m6A antibody, 16 µl 5X IP buffer, 60 µl nuclease-free water) was prepared. Primary antibodies against METTL3 (Abcam monoclonal, Cat# ab195352, 1:1000) and GAPDH (Abcam, monoclonal, Cat# ab125247, 1:1000). Secondary antibodies: Donkey anti-mouse horseradish peroxidase (HRP) (Abcam, Cat# ab6820, 1:3000), donkey anti-rabbit (HRP) (Abcam, Cat# ab6802, 1:3000).
Validation	All antibodies were compared in this study and measured for specificity in comparison to known positive and negative controls based on published data. Data generated for the validation of our method was compared to published state-of-the art data (Liu, J. et al. Landscape and Regulation of m(6)A and m(6)Am Methylome across Human and Mouse Tissues. Mol Cell 77, 426-440 e426 (2020).) For further details, see results and methods sections of the manuscript.

Eukaryotic cell lines

Policy information about cell lines

Cell line source(s)	For Extended Data Fig.7, mES cells (R1) were purchased from American Type Culture Collection (Cat. # SCRC-1011). For Extended Data Fig.4, Mettl3 KO and wild-type control mES cell lines were gifted from Jacob Hanna (Weizmann Institute of Science).
Authentication	Cell lines were authenticated by western blot and RNA-seq.
Mycoplasma contamination	Mycoplasma testing was carried out on a regular basis and all of the cell lines were free for Mycoplasma.
Commonly misidentified lines (See ICLAC register)	No commonly misidentified cell lines were used.

Animals and other organisms

Policy information about studies involving animals; ARRIVE guidelines recommended for reporting animal research

Laboratory animals	C57BL/6N were housed at the Norwegian Transgenic Center (NTS) under 12h:12h light:dark cycles in a specific pathogen-free animal facility at the NTS. Mice had free access to food and water. Mouse oocytes, embryos and liver were collected from C57BL/6N females (4-8 week-old) as described in the Methods section. C57BL/6N males (8-week-old) were used for mating. Using AB wild type female and male zebrafish (6-18 month old), zygotes were collected after fertilization within 30 minutes as described in the Methods section.
Wild animals	No wild animals were used in this study.
Field-collected samples	No field-collected samples were used in this study.
Ethics oversight	Zebrafish and mouse experiments were approved by the Animal Research Committee, the Norwegian Food Safety Authority (NFSA, FOTS ids: #10898 and #24911). Experimental procedures conformed to the ARRIVE guidelines and were conducted in accordance with the ethical guidelines in Directive 2010/63/EU of the European Parliament on the protection of animals used for scientific purposes and Norwegian legislations.

Note that full information on the approval of the study protocol must also be provided in the manuscript.
